# AI Foundation Models for Wearable Movement Data in Mental Health Research

**Published:** 2025-01-14

**Authors:** Franklin Y. Ruan, Aiwei Zhang, Jenny Y. Oh, SouYoung Jin, Nicholas C. Jacobson

**Affiliations:** 1 Center for Technology and Behavioral Health, Geisel School of Medicine, Dartmouth College, Lebanon, NH, United States; 2 Department of Computer Science, Dartmouth College, Hanover, NH, United States; 3 Department of Biomedical Data Science, Geisel School of Medicine, Dartmouth College, Lebanon, NH, United States; 4 Department of Psychiatry, Geisel School of Medicine, Dartmouth College, Lebanon, NH, United States

## Abstract

Pretrained foundation models and transformer architectures have driven the success of large language models (LLMs) and other modern AI breakthroughs. However, similar advancements in health data modeling remain limited due to the need for innovative adaptations. Wearable movement data offers a valuable avenue for exploration, as it’s a core feature in nearly all commercial smartwatches, well established in clinical and mental health research, and the sequential nature of the data shares similarities to language. We introduce the Pretrained Actigraphy Transformer (PAT), the first open source foundation model designed for time-series wearable movement data. Leveraging transformer-based architectures and novel techniques, such as patch embeddings, and pretraining on data from 29,307 participants in a national U.S. sample, PAT achieves state-of-the-art performance in several mental health prediction tasks. PAT is also lightweight and easily interpretable, making it a robust tool for mental health research. GitHub: https://github.com/njacobsonlab/Pretrained-Actigraphy-Transformer/

## 1. Introduction

Movement-based actigraphy, or wearable accelerometer data, was utilized as early as the 1970s to non-intrusively and effectively measure sleep and circadian patterns, particularly in “free-living” (non-laboratory) environments^1–3^. As wearables became more ubiquitous, researchers began to see that actigraphy contained data beyond sleep cycle information; movement intensities throughout time could be correlated to a participant’s behavioral patterns^4–7^. Indeed, developing a fully proportional accelerometer^8^ significantly enhanced the precision of activity monitoring across various settings, facilitating broader applications in psychopathology^9,10^, chronobiology^11,12^, and health risk management^5,13–17^. Presently, wearable sensor data is now a core feature of nearly every commercially available mobile consumer device (e.g. watches, smartphones)^16,18,19^, and actigraphy data has become a rising modality and an important source of health information for clinical researchers^20,21^.

Despite their promise, current actigraphy studies face notable limitations. Historically, actigraphy studies have predominantly relied on simple processing pipelines or traditional statistical feature-based models^20^, and although CNNs and RNN-based LSTMs^22–26^ have been applied to actigraphy, these architectures have inherent limitations. While they excel at capturing short-term patterns, CNNs struggle to identify long-range dependencies critical for time-series data^27–29^. RNN-based LSTMs often face challenges in retaining information over extended time periods due to issues like vanishing gradients, which limit their effectiveness for datasets spanning days or even weeks^30–32^. A significant gap remains between these approaches and the state-of-the-art architectures in fields like natural language processing and computer vision. In these fields, transformers excel at capturing complex, long-range dependencies—features crucial for time-series applications like actigraphy that require modeling interactions across hours or even days^33–35^. To our knowledge, there is no existing foundation model that specifically adapts transformer architectures for wearable accelerometer data. These challenges highlight the need for novel approaches that leverage the strength of transformers while addressing the demands of wearable sensors.

However, adapting traditional transformers to actigraphy is challenging due to the lengthy and dense nature of the data. Transformer models often limit input tokens to the hundreds, as each additional token quadratically increases memory and computation costs^36,37^. For example, one week of actigraphy data produces over 10,000 tokens with minute-level sampling, quickly overwhelming standard transformers. Moreover, many current studies using wearables or actigraphy data tend to feature a limited number of participants^38,39^, as it is often difficult to amass large participant pools for clinical research purposes. Paired with a limited participant size, the complexity of longitudinal actigraphy data poses a challenge for machine learning models, which face a difficult time abstracting from such data. Therefore, foundation models pretrained on extensive datasets offer a promising alternative to training models from scratch. These pretrained models can be fine-tuned on smaller, task-specific actigraphy datasets, leveraging knowledge from broader data sources to achieve robust performance even with limited participant samples.

To address these challenges, we propose designing a simple-to-use yet robust actigraphy foundation model that can be easily deployed for health related studies. First, we construct an attention model architecture for actigraphy. Moving beyond CNNs and LSTM models, we present an Actigraphy Transformer (AT) architecture that utilizes attention mechanisms and patching techniques^40^ to efficiently embed lengthy actigraphy data. Second, we design a mechanism for model explainability. Using attention weights, our model can output visualizations that indicate which minutes of physical activity are most impactful in the model decision. This allows researchers to make reliable inferences about how actigraphy may relate to a health outcome within a study. Third, we pretrain the model in a BERT^41^ or Masked Autoencoder^42^-like manner on actigraphy data from almost 30,000 participants from a US cohort to create the Pretrained Actigraphy Transformer (PAT).

Using datasets where actigraphy is labeled with a participant’s psychotropic medication usage, sleep disorders and abnormalities, or PHQ-9 score (for depression), we aim to evaluate PAT’s efficacy when fine-tuned for mental health studies. We hypothesize that PAT, our explainable foundation model, can deliver state-of-the-art results when fine-tuned for various actigraphy studies, even in datasets containing small amounts of labeled actigraphy data.

## 2. Results

For evaluative purposes, PAT was pretrained on week-long actigraphy data from three nationally representative cohorts of participants in the United States: the NHANES 2003–2004, 2005–2006, and 2011–2012 datasets^43^, totaling 21,538 participants. The fourth cohort, NHANES 2013–2014, consisting of 7,769 participants, was entirely held out in order to create various supervised datasets for testing. Models pretrained on all 29,307 participants, including the 2013–2014 cohort, are also available on our GitHub.

### 2.1 PAT Transfer Learning Results

We held out all NHANES 2013–2014 data to create supervised datasets. Using these datasets, we will evaluate PAT’s ability to predict the following labels: Benzodiazepine medication usage, SSRI medication usage, sleep disorders, sleep abnormalities, and depression. Each label has its own dataset, such that the input is the participant’s actigraphy, and the output is the label (e.g., depression). For each dataset, a test set of 2,000 participants is held out, while the remaining participants are used for training. We compare against several baseline models. These include a logistic regression model trained on wavelet transform features, an LSTM, a 1D CNN, a 3D CNN, and a ConvLSTM. The 3D CNN and ConvLSTM modeling strategies for actigraphy are adapted from Heinz et al.^22^ and Rahman and Adjeroh^25^ and, to our knowledge, are currently the best models used for actigraphy understanding tasks.

#### Predicting benzodiazepine usage.

To examine model performance across varying dataset sizes, we subset participants into groups of “500”, “1,000”, “2,500”, and “5,769” to simulate different levels of data availability. This approach allows us to assess the model’s applicability across scenarios with limited and abundant data, reflecting both personalized and large-scale settings. As seen in Table 1, the best baseline model was the 3D CNN with smoothed actigraphy data, achieving an AUC of 0.677 with 500 participants, 0.695 with 1,000 participants, 0.695 with 2,500 participants, and 0.719 with all 5,769 participants, with an average AUC of 0.697 across all sizes.

PAT models consistently outperform the best baseline. PAT-L scored the best, with an AUC of 0.771 when fine-tuned with only 500 participants, 0.765 with 1,000 participants, 0.760 with 2,500 participants, and 0.771 with all 5,769 participants. The average AUC across all four datasets for PAT-L is 0.767, achieving a 7.0 percentage-point absolute improvement over the best baseline.

#### Predicting SSRI usage, sleep disorder, sleep abnormalities, and depression.

We evaluate our models on predicting SSRI usage, sleep disorders, sleep abnormalities, and depression (see Table 2) in the same way we evaluate our model on predicting benzodiazepine usage, as seen in Table 1.

Most of our PAT models above the small size performed better than the best baseline model found in each dataset. Using the average AUC metric seen in Table 1 and comparing the best PAT model with the best baseline model, we find a 2.0 percentage-point absolute improvement when predicting SSRIs, a 3.2 percentage-point absolute improvement when predicting disorders, a 5.0 percentage-point absolute improvement when predicting sleep abnormalities, and a 2.4 percentage-point absolute improvement when predicting depression. A further breakdown of the model scores can be found in Table 2 and our supplemental information. More information about the datasets can be found in the methods.

### 2.2 PAT Experiments

**Figure 1.**
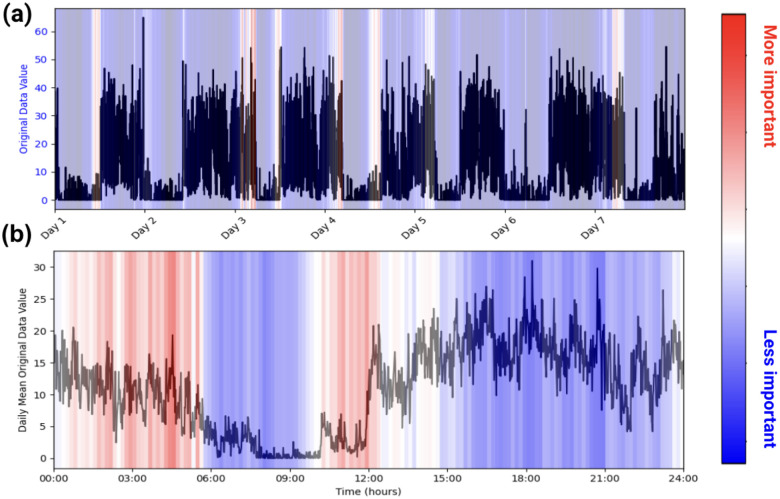
Example of PAT Model Explainability. Plots (a) and (b) are of one participant labeled as taking benzodiazepines. (a) represents the participant’s actigraphy data across a week, and (b) represents that week’s actigraphy data averaged into 1 day. The levels of importance shown in the heatmap are derived from the model’s attention scores during the classification task. Attention scores, which indicate the relative focus of the model on specific time points, were normalized and mapped to a color scale, with red representing regions of higher attention (interpreted as higher importance) and blue indicating regions of lower attention. In this example, the model pays high attention to the behaviors in the early mornings and picks up on the participant’s late (noon) wake-up time when predicting benzodiazepine usage.

We conducted experiments on PAT-M to find the optimal pretraining conditions. We derive all our experiment results from a validation set of 1,000 participants in the benzodiazepine dataset, separate and with no overlap from the held-out test set of the 2,000 participants used for Figure 1.

**Figure 2:**
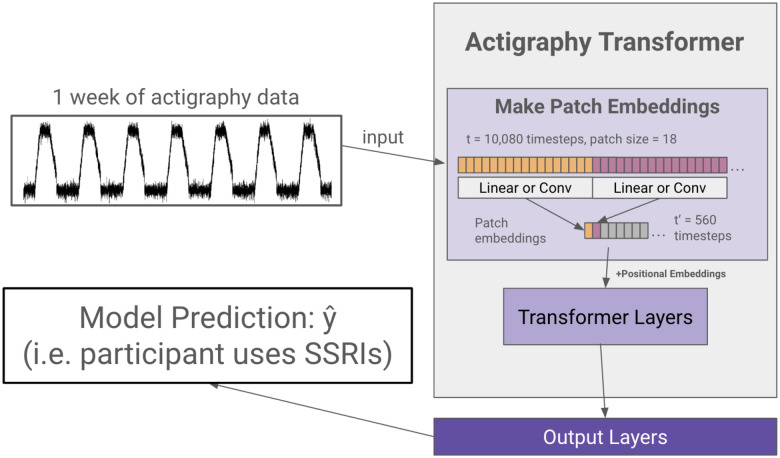
Actigraphy Transformer (Not Pretrained). The figure presents an example case of using an Actigraphy Transformer (AT) for classification. Actigraphy data is fed into the AT and transformed into patches. Assuming a patch size of 18, the number of time steps fed into our transformer is reduced from 10,080 to 560. We add positional embeddings to our patch embeddings before feeding them to the transformer layers. An output layer head is attached to our Actigraphy Transformer and is shown to perform a model classification task.

Table 3 shows that (1) Higher mask ratios during pretraining improve performance; the optimal mask ratio found was 0.90. (2) Smoothing the data does not improve performance. (3) In the reconstruction step of our pretraining, we found that calculating the mean squared error (MSE) for every time step produced a significantly better pretrained model than calculating the MSE only for the masked portions. As such, all PAT models for Figures 1 and 2 are pretrained with these hyperparameters optimized.

### 2.3 Model Explainability

Health-related studies are often interested in the mechanisms behind a phenomenon, but the black-box nature of deep learning models makes it difficult for researchers to characterize associations even if they are found^44,45^. As such, we use the attention weights inherently built into our model to infer which patterns within the accelerometry data are most paid attention to when making a prediction. This allows for easy model explainability, enabling researchers to make characterizations about actigraphy and a given label, as seen in Figure 1.

## 3. Discussion

Accelerometer data from wearables contain a rich source of information for healthcare and research^5,46,47^. Moreover, actigraphy is becoming increasingly relevant as wearable technology becomes more ubiquitous. The easily accessible wearable data, which can be obtained retrospectively and in real-time, shows promise for representing longitudinal and naturalistic behavioral patterns^21,48^.

We note, however, that major improvements can be made to the model architectures currently used for actigraphy studies. Wearable accelerometer data is unique in its ability to provide continuous, minute-level information over long periods, making it invaluable for studying longitudinal behaviors like sleep, activity levels, and mental health outcomes^1,2^. However, the complexity and longitudinal structure of actigraphy data limit feature engineering and traditional machine learning models^23^, as patterns can span across hours or even days, requiring models that capture both short and long term changes such as daily movement and weekly activity cycles, respectively.

The preferred model used for many actigraphy studies, ConvLSTM^24,25,49,50^, is limited by local context, as recurrent and convolutional-based models rely on the inductive bias that data points close together are more related than those further apart. Actigraphy data is often extensive and relies on capturing long-range dependencies and this presents a challenge for the ConvLSTM architecture. To circumvent this, the ConvLSTM model expands the context window for temporal and spatial models by reshaping data into a 3D matrix of 7 days × 24 hours × 60 minutes so that the start of each day is close time-wise, and the start of each hour is close space-wise. Even so, the ConvLSTM may miss certain associations, as researchers introduce strong inductive biases during modeling^51^. For example, the model would struggle to relate events from a Tuesday night to a Thursday morning, as they would continue to be spatially distant. These limitations, driven by inductive biases inherent to recurrent and convolutional architectures, can lead to missed associations when modeling actigraphy data.

We address these issues through our PAT model. Indeed, PAT consistently outperforms the most popular models used for actigraphy in various tasks and at various dataset sizes (Table 1 and Table 2). We develop a transformer-based model because it offers the advantages of global attention^33^, allowing us to extrapolate beyond the local context. For instance, in the context of predicting sleep disorders, our transformers can relate movement patterns at night to a change in behavior in the evening of the next day, or even the day after—despite the fact that these events could be separated by thousands of time steps. Additionally, attention-based models require few inductive priors in contrast to recurrent or convolutional based models; attention-based models extrapolate which minutes of actigraphy data are most related through associations found in the data alone. As such, transformers are more flexible and general, allowing for better embeddings of temporally and spatially complex actigraphy data^33,52^. With PAT, context is elevated to a global scale such that any set of minutes can be associated with any other set of minutes along the week, regardless of spatial or temporal distance.

Another major problem in machine learning and actigraphy-based research is the limited number of participants in many clinical studies^38,39^; it can be difficult for models to abstract information from a limited pool of participants. As such, we pretrained our PAT models on over 20k participants’ unlabeled actigraphy data so that PAT can still produce robust results even when fine-tuned on small amounts of labeled actigraphy data. Table 1 suggests that PAT works well even in limited data conditions. PAT-L shows an 8.8 percentage-point absolute improvement compared to the best baseline model when both were trained on a labeled dataset with only 500 participants.

By leveraging pretrained representations, researchers can reduce the reliance on large labeled datasets, facilitating effective model training across a broader range of clinical scenarios. This robustness makes PATs suitable for studying populations with low base rate diseases or rare conditions, where recruiting sufficient participants can be challenging. In tandem, PATs show promise in personalized or individualized health modeling. Pretrained models like PAT could help address the “cold-start” problem in sensing applications by providing a strong initial model before additional data is collected, or the system becomes actionable.

On the contrary, PATs also show robustness in scaling, which can be important as wearable data becomes more accessible. Natural language processing and computer vision studies have suggested that attention-based models scale better than their convolutional or RNN counterparts^42,52^. Likewise, PAT-L has a 5.2 percentage-point absolute improvement compared to the best baseline model when both models were trained on all available labeled data in Figure 1—and we expect PAT performance to increase with more pretraining data.

Having established the strengths of transformer-based models for actigraphy data, it is equally important to consider how they may be implemented. To increase the model’s accessibility for research, all the code, alongside tutorial notebooks for model explainability, fine-tuning PAT, and pretraining PAT, can be found in our GitHub repository. We also note that our largest model, PAT-L, is just under 2 million parameters. All models can take in variable input lengths, can be developed and trained using accelerators on Google Colab with a Pro account, which costs $10 (USD) per month—a relatively modest expense compared to traditional high-performance computing setups. Each model iteration was able to train in under six hours on Colab Pro, making it feasible for researchers with limited resources to experiment and adapt these models to their specific datasets. Our models are robust yet lightweight and easy to deploy without heavy computational resources in research settings. As wearable technology and longitudinal data become widely available, models like PAT may prove instrumental in harnessing this data to its full clinical and academic capability.

## 4. Methods

### 4.1 Actigraphy Transformer (AT) Architecture

We first create a transformer architecture tailored to embed lengthy actigraphy data (see Figure 2). Using a technique called patching^40,53^, we design our model to efficiently embed long signals or sequences.

#### Model Design.

In the architecture design, we follow the original Transformer^33^ and the Vision Transformer^52^ as closely as possible to benefit from the known scalable nature of computer vision and NLP transformer architectures. A transformer generally receives a 1D sequence of token embeddings as its input, which translates well to the sequential nature of actigraphy data. However, due to the quadratic complexity of the attention mechanism, it is inefficient to input a week’s worth (10,080 minutes) of actigraphy data as individual tokens. Following the Vision Transformer’s and PatchTST’s solution to circumvent the attention’s quadratic complexity in images and lengthy time series, Actigraphy Transformer (AT) breaks the sequence of actigraphy into fixed-sized non-overlapping patches. N=T/S is the number of patches, where T is the total number of time steps, and S is the number of time steps in a patch. The resultant N represents the number of tokens input into AT. Like the Vision Transformer model, the AT attention layer uses a constant vector size D throughout, and we create patch embeddings by mapping each patch to D dimensions using a trainable linear projection. Each AT Transformer block consists of a multiheaded self-attention layer, a layer normalization layer, a feed-forward network, and another layer normalization layer.

#### Positional Embeddings.

Since transformer-based models are inherently permutationally invariant, we add positional embeddings for each patch. We use fixed sine and cosine positional embeddings to retain the location of each patch, which has been shown to work well for transformers in NLP and computer vision^42,52^.

#### Convolutional Patch Embeddings.

As an alternative to creating patch embeddings through linear projections, we also designed an architecture that uses 1D convolutional layers to embed each patch. Models with this embedding have “conv” added to the end of their name.

### 4.2 Pretrained Actigraphy Transformer (PAT)

**Figure 3.**
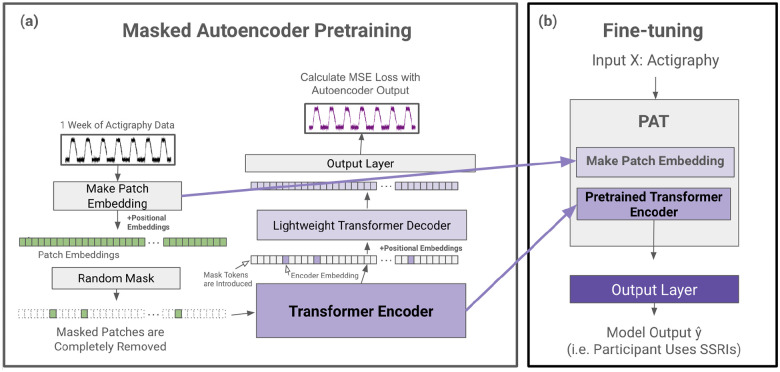
PAT Pretraining and Fine-tuning. (a) During the pretraining phase, actigraphy data is transformed into patch embeddings with positional embeddings and then randomly masked without replacement (e.g. 90% of patches are removed). The remaining patches are fed into a transformer encoder. The encoder embedding tokens are then realigned with masked tokens, and positional embeddings are added again before being fed into a lightweight decoder. The decoder output is then fed to a simple output layer to reconstruct the input actigraphy data. The mean squared error between the input and the reconstructed output is calculated for loss. (b) After pretraining, the patch embedding layer and the pretrained transformer encoder are extracted to create PAT. Any output layer can be added to PAT for a variety of actigraphy understanding tasks (e.g. classification).

Enabled by our Actigraphy Transformer architecture, we utilize a simple masked autoencoder pretraining approach to create deep bidirectional representations of our unlabeled longitudinal actigraphy data (see Figure 3). This way, our pretrained PAT model can be fine-tuned through any labeled actigraphy dataset, including small ones. PAT can then be harnessed to create state-of-the-art models for various applications, such as predicting and characterizing depression, medication use, or sleep disorders through actigraphy data.

#### Masked Autoencoder Design.

Our pretraining task is simple but effective; an encoder creates a latent representation of our input, and a decoder reconstructs the original signal from the encoder representation. We utilize an asymmetric autoencoder similar to the masked autoencoder (MAE) pretraining method used for images^42^. In MAE, random portions of the input data are masked, and the model is trained to reconstruct the missing sections. This process encourages the model to learn meaningful patterns by focusing on context and relationships in the data rather than relying on adjacent information alone. Our encoder encodes only portions of actigraphy data, while our lightweight decoder is tasked with reconstructing an entire week’s actigraphy from the encoder representation tokens and mask tokens. An advantage of our Actigraphy Transformer over conventional convolutional-based models is its ability to divide our sequential data into fixed-size, non-overlapping patches for pretraining. Using this structure, we can randomly select patches to remove or mask once given a mask ratio. As reported in the original MAE paper, we also observed that random masking with a high mask ratio increases the complexity of the reconstruction task, as the autoencoder would not be able to rely merely on adjacent patches of actigraphy for reconstruction (See Table 3).

Following the approach in the original MAE paper, masked patches are entirely removed when fed into the encoder. The pretraining speed is faster since we only feed a small subset of the patches to the encoder. Since our encoder will not encounter mask tokens during the fine-tuning step, removing mask tokens during pretraining has been shown to increase performance^42^. Mask tokens are, however, introduced to the decoder. The input to our decoder, then, is each patch’s embedding from our encoder and mask tokens representing the remaining patches.

As the transformer models are positionally invariant, fixed positional embeddings are added to all the tokens before being inputted into both the encoder and decoder, giving the transformer models information on the position of each token or patch.

#### Reconstruction Target.

Our input into the autoencoder is a sequence of standardized actigraphy data for 10,080 minutes. We attempt to reconstruct this standardized actigraphy data on the minute level with the decoder, such that the output length is also 10,080 minutes. We use a mean squared error at the minute level between the standardized input and the reconstructed output for our loss function.

#### Fine-tuning.

We remove and extract the weights of our patch embedding layer and encoder for the fine-tuning step. We name this encoder and its layer for creating patch embeddings Pretrained Actigraphy Transformer (PAT). Any layers can be added to the end of PAT for differing tasks. For our evaluation purposes, we add a small feed forward layer and a sigmoid activation function at the end of PAT for binary classification. During fine-tuning, we evaluate end-to-end fine-tuning (FT), where we freeze no weights, and linear probing (LP), where we freeze PAT and only train the added layers. Since FT and LP yielded similar results, we only report FT in this manuscript, with LP results shown in the supplementals.

##### Model Explainability

We use our models’ intrinsic attention matrices for simple model explainability. We extract the attention weights from the last transformer block since that block generally yields the most abstracted data^33^. From there, we sum the attention weights across the key positions for each query. Then, we average and normalize the summed attention weights across each head. Using this method, we can see which patches (representing short sequences of activity) our model pays the most attention to. See Figure 1.

### 4.3 Data and Evaluation Scheme

#### Data Source and Collection

This study utilizes participant data from the National Health and Nutrition Examination Survey (NHANES)^43^, which has received approval from the NCHS Research Ethics Review Board. NHANES is a longitudinal, nationally representative study designed to assess the health and nutritional status of the American population. Actigraphy data was collected from differing individuals during the study years 2003–2004, 2005–2006, 2011–2012, and 2013–2014. From 2003 to 2006, data was captured using a hip-mounted ActiGraph AM-7164 piezoelectric accelerometer, which records uniaxial acceleration as summed activity intensity per minute over a week. In later cycles from 2011 to 2014, NHANES shifted to a wrist-worn triaxial accelerometer (Actigraph GT3X+). We use the summed triaxial data per minute to align with earlier uniaxial data.

#### Pretraining Data.

The pretraining task requires only the actigraphy data. The 2003–2004 dataset contains actigraphy data for 7,176 participants, the 2005–2006 dataset contains actigraphy data for 7,455 participants, and the 2011–2012 actigraphy dataset contains actigraphy data for 6,907 participants. In total, data from 21,538 participants was used for pretraining. Each participant in this study recorded one week or 10,080 minutes of accelerometer data, and each dataset is standardized with respect to itself.

A model pretrained on every year of available actigraphy data, including 2013–2014, for a total of 29,307 participants, is also available on our GitHub, though it has not been evaluated.

#### Supervised Training Dataset.

We used the 2013–2014 NHANES dataset for the supervised training tasks. This data, collected via wrist-worn accelerometers, differs from the hip-mounted devices used in the earlier pretraining phase. The focus of the 2013 dataset is to assess the model’s predictive performance on various health outcomes, including benzodiazepine usage, depression, sleep disorders, and sleep abnormalities, based on actigraphy data.

A subset of 7,769 participants who provided actigraphy and medication data was identified. Using this data, we create the benzodiazepine and SSRI datasets. In the benzodiazepine dataset, we label participants taking benzodiazepines as 1 and all others as 0. In the SSRI dataset, we label participants taking SSRIs as 1 and all others as 0. A subset of 4,800 participants had accompanying PHQ-9 depression screener^54,55^ results alongside actigraphy data. We create a dataset that labels participants with a PHQ-9 score of 10 or above as 1 (has depression) and all others as 0. A subset of 5,429 participants had actigraphy and sleep disorder data, as per the SLQ-sleep survey. Using this data, we create two datasets: sleep disorders and sleep abnormalities. For the sleep disorder dataset, if the participant reports having had a sleep disorder at any time in their life, they were labeled as 1 and all others as 0. For the sleep abnormalities dataset, participants were labeled 1 if they had any of the following: (1) had a sleep disorder at any time in their life, (2) regularly slept more than or equal to 12 hours, or (3) regularly slept less than or equal to 5 hours.

#### Supervised Learning Evaluation Scheme.

Each dataset is first split into a train set and then a held-out test set with 2,000 participants. For any dataset, assume N is the total number of participants left in the training set. To assess a model’s ability to train on various dataset sizes, we sample participants with replacement using a stratified splitting method and create the following test sizes: “500”, “1,000”, “2,500”, and N. We then take 20% of each new dataset to create corresponding validation sets. We save all these new datasets to train our model.

#### Evaluation Metric.

We use AUC, or the area under the receiver operating characteristic curve, to evaluate our model’s performance in binary classification. To assess our model’s performance, even when the presented supervised training data is limited, we rank our models after averaging their AUC scores on the test set after being trained on each dataset size “500”, “1,000”, “2,500”, and N. A higher average AUC score is better.

#### PAT Experiments.

We perform experiments on the best pretraining parameters using only the benzodiazepine dataset. With 7,769 participants, 2,000 were first removed to create a held-out test set. The remaining 5,769 participants were used to find the optimal parameters for pretraining, including the optimal mask ratio, loss function, and whether or not using smoothed data bolsters performance. 1,000 participants were set aside for this experiment, distinct from the held-out test set. This left us with 4,769 participants with whom to train the model. We searched for the best pretraining parameters using the evaluation metric mentioned in the section above (i.e., the best average AUC score on the 1,000-participants used for this experiment).

#### Smoothing and Standardization.

In models where we trained with smoothed data, we used a Savitzky-Golay filter with a window length of 51-time steps and a polynomial order of 3, which has been shown to retain temporal patterns while reducing noise^23,56^.

In our supervised datasets, all train, test, and validation sets were standardized separately using Sklearn’s StandardScaler, such that each activity intensity is reported as a standard deviation from the mean of a given minute in the sequence. In our pretraining data, we standardized the 2003–2004, 2005–2006, and 2011–2012 datasets separately, as differing devices were used throughout the years. Standardization allows consistency among actigraphy datasets used, where absolute accelerometer measurements may vary.

## Supplementary Material

1

## Figures and Tables

**Table 1:** Evaluating models on the ability to predict benzodiazepine usage from actigraphy. In this dataset, the input is actigraphy and the label indicates whether a participant is taking benzodiazepines. Each model is trained on dataset sizes “500”, “1,000”, “2,500”, and “5,769” (seen in the columns) and evaluated using AUC on a held-out test set of 2,000 participants. The “Avg AUC” represents the averaged AUC scores across each training dataset size. If the model name has “smoothing” after it, it denotes that the model was trained on smoothed data. Underlined text indicates the best baseline model. PAT-S/M/L denotes Small, Medium, Large. A bolded PAT model indicates that it performed better than the best baseline, and a bolded and underlined PAT indicates the model with the best performance. PATs significantly outperform the baseline models in every dataset size in this task.

MODEL	AvgAUC*	n=500	n=1000	n=2500	n=5769	Params
LSTM	0.493	0.501	0.487	0.474	0.512	15 K
LSTM (smoothing)	0.499	0.506	0.508	0.482	0.499	15 K
Wavelet Transform	0.620	0.674	0.625	0.598	0.583	10 K
CNN-1D	0.632	0.621	0.630	0.640	0.637	10 K
CNN-1D (smoothing)	0.639	0.633	0.634	0.644	0.646	10 K
Conv LSTM (smoothing)	0.667	0.666	0.680	0.653	0.671	1.75 M
Conv LSTM	0.668	0.663	0.681	0.650	0.677	1.75 M
CNN-3D	0.693	0.683	0.693	0.693	0.703	790 K
CNN-3D (smoothing)	0.697	0.677	0.695	0.696	0.719	790 K
**PAT-S**	**0.701**	0.706	0.718	0.677	0.703	285 K
**PAT Conv-S**	**0.726**	0.737	0.711	0.722	0.735	285 K
**PAT-M**	**0.744**	0.743	0.745	0.742	0.745	1.00M
**PAT Conv-M**	**0.761**	0.753	0.756	0.760	0.773	1.00M
**PAT Conv-L**	**0.762**	0.763	0.756	0.754	0.773	1.99M
**PAT-L**	**0.767**	0.771	0.765	0.760	0.771	1.99M

**Table 2:** Model performance across different actigraphy tasks (predicting SSRI usage, Sleep Disorder, Sleep Abnormalities, and Depression). The table summarizes the performance of our PAT models versus various baseline models (including LSTM, CNN, ConvLSTM, and 3D CNN) across four tasks: predicting SSRI usage, history of any sleep disorder, abnormal sleep patterns, and depression. Each model is trained on dataset sizes “500”, “1,000”, “2,500”, and all available data (5,769 for SSRI usage, 3,429 for Sleep Disorder and Sleep abnormalities, and 2,800 for Depression) and evaluated using AUC on a held-out test set of 2,000 participants. The score for each model here represents the averaged AUC scores across each training dataset size. If the model name has “smoothing” after it, it denotes that the model was trained on smoothed data. An underline indicates the best baseline model. PAT-S/M/L denotes Small, Medium, Large. A bolded PAT model indicates that it performed better than the best baseline, and a bolded and underlined PAT indicates the model with the best performance. The results suggest that PATs outperform baseline models in various actigraphy understanding tasks and at various dataset sizes.

MODEL	SSRI usage	Sleep Disorder	Sleep Abnormalities	Depression	Params
LSTM	0.527	0.494	0.513	0.489	15 K
LSTM (smoothing)	0.523	0.506	0.515	0.506	15 K
Wavelet Transform	0.674	0.529	0.525	0.523	10 K
CNN-1D	0.616	0.563	0.534	0.522	10 K
CNN-1D (smoothing)	0.611	0.558	0.519	0.517	10 K
Conv LSTM (smoothing)	0.655	0.609	0.579	0.547	1.75 M
Conv LSTM	0.606	0.606	0.585	0.550	1.75 M
CNN-3D	0.677	0.608	0.606	0.583	790 K
CNN-3D (smoothing)	0.680	0.605	0.615	0.586	790 K
PAT-S	0.641	0.587	0.555	0.560	285 K
PAT Conv-S	0.656	**0.616**	0.573	**0.587**	285 K
PAT-M	**0.690**	**0.641**	**0.641**	0.559	1.00M
PAT Conv-M	0.668	**0.616**	**0.627**	**0.594**	1.00M
PAT Conv-L	**0.695**	**0.631**	**0.659**	**0.610**	1.99M
PAT-L	**0.700**	**0.632**	**0.665**	**0.589**	1.99M

**Table 3:** PAT-M Experiments. All models are pretrained and then end-to-end fine-tuned on the benzodiazepine training data. For the experiments, 1,000 participants were removed from the training data to create an evaluation set, and these 1,000 participants are separate from the held-out test set seen in Table 1. The “Avg Score” metric is the average AUC score on the evaluation set after the medium model was trained on dataset sizes “500”, “1,000”, “2,500”, and “4,769”. (a) We tested a PAT-M pretrained using MSE loss on every data point. We found that a higher mask ratio during pretraining leads to better results. (b) We tested PAT-M pretrained on 90% masking and MSE loss on all data and found that smoothing does not improve performance. (c) We tested a PAT-M with 90% masking and found that MSE on only the masked patches decreased performance drastically.

(a) MODEL (Mask Ratio)	Avg Score*
Medium 0.25	0.737
Medium 0.50	0.707
Medium 0.75	0.743
**Medium 0.90**	**0.773**

(b) MODEL (Smoothing)	Avg Score*	(c) MODEL (Loss Function)	Avg Score*
Medium (Smoothed)	0.741	Medium (MSE Only Masked)	0.541
**Medium (Not Smooth)**	**0.773**	**Medium (MSE All)**	**0.773**

## Data Availability

The datasets used during the current study are available from the National Health and Nutrition Examination Survey (NHANES), maintained by the Centers for Disease Control and Prevention (CDC). These datasets are publicly available and can be accessed at https://wwwn.cdc.gov/nchs/nhanes/Default.aspx.
