# Supplementary material for: AI Foundation Models for Wearable Movement Data in Mental Health Research
Source: ArXiv. 2025 Jan 14:arXiv:2411.15240v3. Preprint. [Version 3] (PMC11623705)
Supplement: Supplementary file 1 [file NIHPP2411.15240V3-supplement-1.pdf]

Supplementary Material

Supplemental Table 1. Evaluating models predicting benzodiazepine usage from actigraphy (Including linear probing data).

MODEL	Avg AUC*	n=500	n=1000	n=2500	n=5769	Params
LSTM	0.493	0.501	0.487	0.474	0.512	15 K
LSTM (smoothing)	0.499	0.506	0.508	0.482	0.499	15 K
Wavelet Transform	0.620	0.674	0.625	0.598	0.583	10 K
CNN-1D	0.632	0.621	0.630	0.640	0.637	10 K
CNN-1D (smoothing)	0.639	0.633	0.634	0.644	0.646	10 K
Conv LSTM (smoothing)	0.667	0.666	0.680	0.653	0.671	1.75 M
Conv LSTM	0.668	0.663	0.681	0.650	0.677	1.75 M
CNN-3D	0.693	0.683	0.693	0.693	0.703	790 K
CNN-3D (smoothing)	0.697	0.677	0.695	0.696	0.719	790 K
**PAT-S (FT)**	**0.701**	0.706	0.718	0.677	0.703	285 K
**PAT-S (LP)**	**0.706**	0.721	0.721	0.677	0.705	285 K
**PAT Conv-S (FT)**	**0.726**	0.737	0.711	0.722	0.735	285 K
**PAT Conv-S (LP)**	**0.727**	0.734	0.713	0.722	0.738	285 K
**PAT-M (FT)**	**0.744**	0.743	0.745	0.742	0.745	1.00 M
**PAT-M (LP)**	**0.745**	0.734	0.746	0.742	0.756	1.00 M
**PAT Conv-M (LP)**	**0.759**	0.753	0.753	0.759	0.773	1.00 M
**PAT Conv-M (FT)**	**0.761**	0.753	0.756	0.760	0.773	1.00 M
**PAT Conv-L (FT)**	**0.762**	0.763	0.756	0.754	0.773	1.99 M
**PAT Conv-L (LP)**	**0.762**	0.765	0.755	0.754	0.773	1.99 M
**PAT-L (FT)**	**0.767**	0.771	0.765	0.760	0.771	1.99 M
**PAT-L (LP)**	**0.768**	0.775	0.764	0.760	0.771	1.99 M

Supplemental Table 1 Evaluating models predicting benzodiazepine usage from actigraphy. *The difference between this table and Table 1 in the manuscript is that we include linear probing data, denoted as LP. FT stands for end-to-end finetuning. In this dataset, the input is actigraphy, and the label indicates whether that participant is taking benzodiazepines. Each model is trained on dataset sizes "500", "1,000", "2,500", and "5,769", (seen in the columns) and evaluated using AUC on a held-out test set of 2,000 participants. The "Avg AUC" represents the averaged AUC scores across each training dataset size. If the model name has "smoothing" after it, it denotes that it was trained on smoothed data. An underline indicates the best baseline model. A bolded PAT model suggests that it performed better than the best baseline, and a bolded and underlined PAT indicates the model with the best performance. PATs significantly outperform the baseline models in every dataset size in this task.*

Supplemental Table 2. Evaluating models predicting SSRI usage from actigraphy.

MODEL	Avg Score*	n=500	n=1000	n=2500	n=5769	Params
LSTM (Smooth)	0.523	0.520	0.505	0.541	0.527	15 K
LSTM	0.527	0.533	0.534	0.518	0.524	15 K
Wavelet Transform	0.572	0.569	0.559	0.552	0.606	10 K
Conv LSTM	0.606	0.444	0.585	0.691	0.703	1.75 M
CNN-1D (Smooth)	0.611	0.487	0.643	0.651	0.664	10 K
CNN-1D	0.616	0.548	0.600	0.660	0.655	10 K
PAT-S (FT)	0.641	0.586	0.626	0.674	0.679	285 K
PAT-S (LP)	0.643	0.598	0.617	0.676	0.679	285 K
Conv LSTM (Smooth)	0.655	0.583	0.639	0.700	0.698	1.75 M
PAT Conv-S (FT)	0.656	0.536	0.711	0.692	0.684	285 K
PAT-L (LP)	0.662	0.495	0.721	0.713	0.720	1.99 M
PAT Conv-S (LP)	0.666	0.571	0.714	0.693	0.687	285 K
PAT Conv-M (FT)	0.668	0.552	0.705	0.705	0.712	1.00 M
CNN-3D	0.677	0.668	0.678	0.668	0.695	790 K
CNN-3D (Smooth)	0.680	0.671	0.675	0.682	0.692	790 K
**PAT Conv-M (LP)**	**0.680**	0.597	0.707	0.703	0.714	1.00 M
**PAT-M (FT)**	**0.690**	0.661	0.704	0.684	0.710	1.00 M
**PAT Conv-L (LP)**	**0.694**	0.674	0.718	0.709	0.674	1.99 M
**PAT Conv-L (FT)**	**0.695**	0.677	0.717	0.710	0.675	1.99 M
**PAT-L (FT)**	**0.700**	0.651	0.720	0.710	0.721	1.99 M
**PAT-M (LP)**	**0.702**	0.698	0.702	0.699	0.710	1.00 M

Supplemental Table 2 Evaluating models predicting SSRI usage from actigraphy. *In this dataset, the input is actigraphy, and the label indicates whether that participant is taking SSRIs. Each model is trained on dataset sizes "500", "1,000", "2,500", and "5,769", (seen in the columns) and evaluated using AUC on a held-out test set of 2,000 participants. The "Avg AUC" represents the averaged AUC scores across each training dataset size. If the model name has "smoothing" after it, it denotes that it was trained on smoothed data. LP stands for linear probing, and FT stands for end-to-end finetuning. An underline indicates the best baseline model. A bolded PAT model suggests that it performed better than the best baseline, and a bolded and underlined PAT indicates the model with the best performance. PATs outperform the baseline models in every dataset size in this task.*

Supplemental Table 3. Evaluating models predicting if a participant has or once had a sleep disorder from actigraphy.

MODEL	Avg Score*	n=500	n=1000	n=2500	n=3429	Params
LSTM	0.494	0.480	0.490	0.509	0.499	15 K
LSTM (Smooth)	0.506	0.486	0.511	0.489	0.540	15 K
Wavelet Transform	0.529	0.525	0.510	0.544	0.539	10 K
CNN-1D (Smooth)	0.558	0.556	0.540	0.570	0.566	10 K
CNN-1D	0.563	0.571	0.545	0.568	0.569	10 K
PAT-S (FT)	0.587	0.584	0.546	0.605	0.612	285 K
PAT-S (LP)	0.596	0.586	0.579	0.607	0.613	285 K
CNN-3D (Smooth)	0.605	0.600	0.597	0.601	0.621	790 K
Conv LSTM	0.606	0.591	0.608	0.602	0.623	1.75 M
CNN-3D	0.608	0.611	0.612	0.587	0.624	790 K
Conv LSTM (Smooth)	0.609	0.591	0.604	0.609	0.633	1.75 M
**PAT Conv-S (LP)**	**0.613**	0.600	0.619	0.615	0.620	285 K
**PAT Conv-M (FT)**	**0.616**	0.588	0.622	0.617	0.637	1.00 M
**PAT Conv-S (FT)**	**0.616**	0.600	0.624	0.617	0.622	285 K
**PAT Conv-M (LP)**	**0.616**	0.587	0.621	0.618	0.637	1.00 M
**PAT Conv-L (LP)**	**0.627**	0.614	0.632	0.626	0.636	1.99 M
**PAT Conv-L (FT)**	**0.631**	0.624	0.633	0.630	0.637	1.99 M
**PAT-L (FT)**	**0.632**	0.633	0.644	0.613	0.638	1.00 M
**PAT-L (LP)**	**0.634**	0.631	0.644	0.621	0.639	1.00 M
**PAT-M (FT)**	**0.641**	0.625	0.647	0.639	0.652	1.99 M
**PAT-M (LP)**	**0.641**	0.624	0.647	0.640	0.652	1.99 M

Supplemental Table 3 Evaluating models predicting if participant has or once had a sleep disorder from actigraphy. *In this dataset, the input is actigraphy, and the label indicates if a participant has or once had a sleep disorder. Each model is trained on dataset sizes "500", "1,000", "2,500", and "3,429", (seen in the columns) and evaluated using AUC on a held-out test set of 2,000 participants. The "Avg AUC" represents the averaged AUC scores across each training dataset size. If the model name has "smoothing" after it, it denotes that it was trained on smoothed data. LP stands for linear probing, and FT stands for end-to-end finetuning. An underline indicates the best baseline model. A bolded PAT model suggests that it performed better than the best baseline, and a bolded and underlined PAT indicates the model with the best performance. PATs outperform the baseline models in every dataset size in this task.*

Supplemental Table 4. Evaluating models in predicting sleep abnormality from actigraphy.

MODEL	Avg Score*	n=500	n=1000	n=2500	n=3429	Params
LSTM	0.513	0.500	0.498	0.532	0.524	15 K
LSTM (Smooth)	0.515	0.522	0.524	0.493	0.522	15 K
CNN-1D (Smooth)	0.519	0.478	0.513	0.534	0.550	10 K
Wavelet Transform	0.525	0.503	0.525	0.547	0.524	10 K
CNN-1D	0.534	0.501	0.548	0.549	0.538	10 K
PAT-S (FT)	0.555	0.527	0.516	0.610	0.568	285 K
PAT-S (LP)	0.565	0.531	0.558	0.607	0.565	285 K
PAT Conv-S (LP)	0.571	0.512	0.596	0.610	0.564	285 K
PAT Conv-S (FT)	0.573	0.506	0.604	0.615	0.568	285 K
Conv LSTM (Smooth)	0.579	0.518	0.609	0.592	0.598	1.75 M
Conv LSTM	0.585	0.558	0.607	0.586	0.589	1.75 M
CNN-3D	0.606	0.596	0.632	0.547	0.650	790 K
CNN-3D (Smooth)	0.615	0.588	0.618	0.628	0.625	790 K
**PAT Conv-M (FT)**	**0.627**	0.591	0.624	0.649	0.644	1.00 M
**PAT Conv-M (LP)**	**0.632**	0.599	0.632	0.649	0.647	1.00 M
**PAT-M (LP)**	**0.641**	0.583	0.653	0.661	0.665	1.00 M
**PAT-M (FT)**	**0.641**	0.585	0.653	0.661	0.666	1.00 M
**PAT Conv-L (LP)**	**0.659**	0.614	0.661	0.676	0.685	1.99 M
**PAT Conv-L (FT)**	**0.659**	0.616	0.661	0.675	0.685	1.99 M
**PAT-L (LP)**	**0.665**	0.627	0.667	0.678	0.686	1.99 M
**PAT-L (FT)**	**0.665**	0.626	0.667	0.679	0.686	1.99 M

Supplemental Table 4 Evaluating models in predicting sleep abnormality from actigraphy. *In this dataset, the input is actigraphy, and the label indicates whether that participant has sleep abnormalities. Each model is trained on dataset sizes "500", "1,000", "2,500", and "3,429", (seen in the columns) and evaluated using AUC on a held-out test set of 2,000 participants. The "Avg AUC" represents the averaged AUC scores across each training dataset size. If the model name has "smoothing" after it, it denotes that it was trained on smoothed data. LP stands for linear probing, and FT stands for end-to-end finetuning. An underline indicates the best baseline model. A bolded PAT model suggests that it performed better than the best baseline, and a bolded and underlined PAT indicates the model with the best performance. PATs outperform the baseline models in every dataset size in this task.*

Supplemental Table 5. Evaluating models predicting depression from actigraphy.

MODEL	Avg Score*	n=500	n=1000	n=2500	n=2800	Params
LSTM	0.489	0.472	0.489	0.497	0.497	15 K
LSTM (Smooth)	0.506	0.496	0.494	0.519	0.515	15 K
CNN-1D (Smooth)	0.517	0.461	0.540	0.537	0.528	10 K
CNN-1D	0.522	0.500	0.533	0.530	0.525	10 K
Wavelet Transform	0.523	0.550	0.531	0.512	0.500	10 K
Conv LSTM (Smooth)	0.547	0.476	0.561	0.573	0.580	1.75 M
Conv LSTM	0.550	0.507	0.534	0.579	0.581	1.75 M
PAT-M (LP)	0.557	0.488	0.597	0.564	0.577	1.00 M
PAT-M (FT)	0.559	0.489	0.591	0.566	0.589	1.00 M
PAT-S (LP)	0.560	0.547	0.552	0.565	0.575	285 K
PAT-S (FT)	0.560	0.550	0.556	0.560	0.574	285 K
PAT-L (LP)	0.582	0.495	0.595	0.618	0.620	1.99 M
CNN-3D (Smooth)	0.583	0.576	0.576	0.593	0.589	790 K
<u>CNN-3D</u>	<u>0.586</u>	<u>0.587</u>	<u>0.580</u>	<u>0.598</u>	<u>0.580</u>	<u>790 K</u>
**PAT Conv-S (FT)**	**0.587**	**0.568**	**0.576**	**0.603**	**0.600**	**285 K**
**PAT Conv-S (LP)**	**0.587**	**0.567**	**0.575**	**0.604**	**0.603**	**285 K**
**PAT-L (FT)**	**0.589**	**0.541**	**0.577**	**0.618**	**0.620**	**1.99 M**
**PAT Conv-M (LP)**	**0.589**	**0.556**	**0.584**	**0.611**	**0.605**	**1.00 M**
**PAT Conv-M (FT)**	**0.594**	**0.576**	**0.585**	**0.609**	**0.606**	**1.00 M**
**PAT Conv-L (FT)**	**0.610**	**0.594**	**0.606**	**0.617**	**0.624**	**1.99 M**
**PAT Conv-L (LP)**	**0.611**	**0.594**	**0.606**	**0.618**	**0.625**	**1.99 M**

Supplemental Table 5 Evaluating models predicting depression from actigraphy. *In this dataset, the input is actigraphy, and the label indicates whether that participant has depression (PHQ-9 scores > 9). Each model is trained on dataset sizes "500", "1,000", "2,500", and "2,800", (seen in the columns) and evaluated using AUC on a held-out test set of 2,000 participants. The "Avg AUC" represents the averaged AUC scores across each training dataset size. If the model name has "smoothing" after it, it denotes that it was trained on smoothed data. LP stands for linear probing, and FT stands for end-to-end finetuning. An underline indicates the best baseline model. A bolded PAT model suggests that it performed better than the best baseline, and a bolded and underlined PAT indicates the model with the best performance. PATs outperform the baseline models in every dataset size in this task.*

Supplemental Table 6. Finding optimizing mask ratio, data preprocessing, and loss function.

(a) MODEL (Size, Masking Ratio)	Avg Score*	n=500	n=1000	n=2500	n=4769
Medium 0.50 (LP)	0.703	0.661	0.672	0.693	0.785
Medium 0.50 (FT)	0.707	0.727	0.672	0.642	0.788
Medium 0.25 (LP)	0.724	0.689	0.698	0.765	0.744
Medium 0.25 (FT)	0.737	0.711	0.734	0.745	0.759
Medium 0.75 (FT)	0.743	0.662	0.742	0.798	0.772
Medium 0. 75 (LP)	0.747	0.686	0.753	0.781	0.767
Medium 0.90 (LP)	0.768	0.720	0.766	0.794	0.792
**Medium 0.90 (FT)**	**0.773**	0.753	0.764	0.786	0.788

(b) MODEL (Size, Smoothing)	Avg Score*	n=500	n=1000	n=2500	n=4769
Medium Smooth (LP)	0.734	0.686	0.686	0.767	0.797
Medium Smooth (FT)	0.741	0.732	0.682	0.756	0.792
Medium Raw (LP)	0.768	0.720	0.766	0.794	0.792
**Medium Raw (FT)**	**0.773**	0.753	0.764	0.786	0.788

(c) MODEL (Size, Loss)	Avg Score*	n=500	n=1000	n=2500	n=4769
Medium, MSE MASK (LP)	0.534	0.461	0.430	0.542	0.704
Medium, MSE MASK (FT)	0.541	0.437	0.515	0.560	0.652
Medium, MSE ALL (LP)	0.768	0.720	0.766	0.794	0.792
**Medium, MSE ALL (FT)**	**0.773**	0.753	0.764	0.786	0.788

Supplemental Table 6 Finding optimizing mask ratio, data preprocessing, and loss function. *All models are pretrained and end-to-end fine-tuned (FT) on the benzodiazepine training data. The difference between this table and Table 7 in the manuscript is that we also show linear probing (LP) results. For the experiments, 1,000 participants were removed from the training data to create an evaluation set, and these 1,000 participants are separate from the held-out test set seen in Table 1. The "Avg Score" metric is the average AUC score on the evaluation set after the medium model was trained on dataset sizes "500", "1,000", "2,500", and "4,769". (a) We test a PAT-M pretrained using MSE loss on every data point. We find that a higher mask ratio during pretraining leads to better results. (b) We test PAT-M pretrained on 90% masking and MSE loss on all data and find that smoothing does not improve performance (c) We test a PAT-M with 90% masking and find that MSE on only the masked patches decreases performance.***

Supplemental Figure 1: Attention Weight Patterns and Daily Activity Trends for a Non-Benzodiazepine Participant

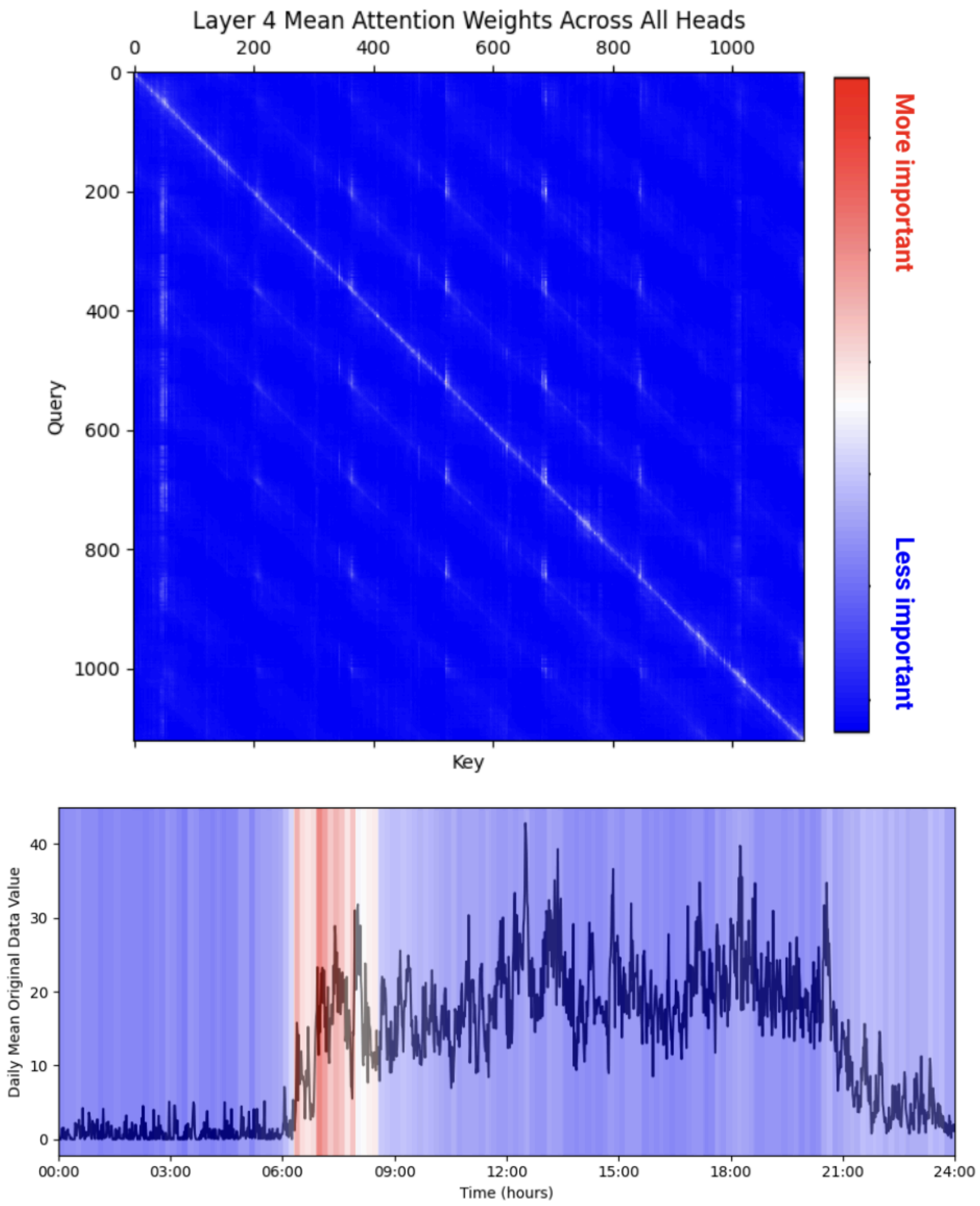

Supplemental Figure 1. Attention Weight Patterns and Daily Actigraphy Trends for a Non-Benzodiazepine Participant. *This figure presents attention weight patterns and daily actigraphy trends for a participant who is not taking benzodiazepines. (Top Panel) The attention weight matrix from Layer 4 of the PAT model across all heads. Bright white dots and the diagonals between them correspond to naturally captured relationships such as consecutive days and hours. (Bottom Panel) Daily mean actigraphy values, with attention weights mapped to activity levels. The model highlights early and sharp wake-up times as important features deciding whether or not a participant takes benzodiazepines.*
