# Supplementary material for: Is Attention All You Need For Actigraphy? Foundation Models of Wearable Accelerometer Data for Mental Health Research
Source: ArXiv. 2024 Nov 26:arXiv:2411.15240v2. Preprint. [Version 2] (PMC11623705)
Supplement: Supplementary file 1 [file NIHPP2411.15240v2-supplement-1.pdf]

# Supplementary Material

**Supplemental Table 1. Evaluating models predicting benzodiazepine usage from actigraphy (Including linear probing data).**

| MODEL | Avg AUC* | n=500 | n=1000 | n=2500 | n=5769 | Params |
|---|---|---|---|---|---|---|
| LSTM | 0.493 | 0.501 | 0.487 | 0.474 | 0.512 | 15 K |
| LSTM (smoothing) | 0.499 | 0.506 | 0.508 | 0.482 | 0.499 | 15 K |
| Wavelet Transform | 0.620 | 0.674 | 0.625 | 0.598 | 0.583 | 10 K |
| CNN-1D | 0.632 | 0.621 | 0.630 | 0.640 | 0.637 | 10 K |
| CNN-1D (smoothing) | 0.639 | 0.633 | 0.634 | 0.644 | 0.646 | 10 K |
| Conv LSTM (smoothing) | 0.667 | 0.666 | 0.680 | 0.653 | 0.671 | 1.75 M |
| Conv LSTM | 0.668 | 0.663 | 0.681 | 0.650 | 0.677 | 1.75 M |
| CNN-3D | 0.693 | 0.683 | 0.693 | 0.693 | 0.703 | 790 K |
| CNN-3D (smoothing) | 0.697 | 0.677 | 0.695 | 0.696 | 0.719 | 790 K |
| **PAT-S (FT)** | **0.701** | 0.706 | 0.718 | 0.677 | 0.703 | 285 K |
| **PAT-S (LP)** | **0.706** | 0.721 | 0.721 | 0.677 | 0.705 | 285 K |
| **PAT Conv-S (FT)** | **0.726** | 0.737 | 0.711 | 0.722 | 0.735 | 285 K |
| **PAT Conv-S (LP)** | **0.727** | 0.734 | 0.713 | 0.722 | 0.738 | 285 K |
| **PAT-M (FT)** | **0.744** | 0.743 | 0.745 | 0.742 | 0.745 | 1.00 M |
| **PAT-M (LP)** | **0.745** | 0.734 | 0.746 | 0.742 | 0.756 | 1.00 M |
| **PAT Conv-M (LP)** | **0.759** | 0.753 | 0.753 | 0.759 | 0.773 | 1.00 M |
| **PAT Conv-M (FT)** | **0.761** | 0.753 | 0.756 | 0.760 | 0.773 | 1.00 M |
| **PAT Conv-L (FT)** | **0.762** | 0.763 | 0.756 | 0.754 | 0.773 | 1.99 M |
| **PAT Conv-L (LP)** | **0.762** | 0.765 | 0.755 | 0.754 | 0.773 | 1.99 M |
| **PAT-L (FT)** | **0.767** | 0.771 | 0.765 | 0.760 | 0.771 | 1.99 M |
| **PAT-L (LP)** | **0.768** | 0.775 | 0.764 | 0.760 | 0.771 | 1.99 M |

***Supplemental Table 1 Evaluating models predicting benzodiazepine usage from actigraphy.*** *The difference between this table and Table 1 in the manuscript is that we include linear probing data, denoted as LP. FT stands for end-to-end finetuning. In this dataset, the input is actigraphy, and the label indicates whether that participant is taking benzodiazepines. Each model is trained on dataset sizes "500", "1,000", "2,500", and "5,769", (seen in the columns) and evaluated using AUC on a held-out test set of 2,000 participants. The "Avg AUC" represents the averaged AUC scores across each training dataset size. If the model name has "smoothing" after it, it denotes that it was trained on smoothed data. An underline indicates the best baseline model. A bolded PAT model suggests that it performed better than the best baseline, and a bolded and underlined PAT indicates the model with the best performance. PATs significantly outperform the baseline models in every dataset size in this task.*

**Supplemental Table 2. Evaluating models predicting SSRI usage from actigraphy.**

| MODEL | Avg Score* | n=500 | n=1000 | n=2500 | n=5769 | Params |
|---|---|---|---|---|---|---|
| LSTM (Smooth) | 0.523 | 0.520 | 0.505 | 0.541 | 0.527 | 15 K |
| LSTM | 0.527 | 0.533 | 0.534 | 0.518 | 0.524 | 15 K |
| Wavelet Transform | 0.572 | 0.569 | 0.559 | 0.552 | 0.606 | 10 K |
| Conv LSTM | 0.606 | 0.444 | 0.585 | 0.691 | 0.703 | 1.75 M |
| CNN-1D (Smooth) | 0.611 | 0.487 | 0.643 | 0.651 | 0.664 | 10 K |
| CNN-1D | 0.616 | 0.548 | 0.600 | 0.660 | 0.655 | 10 K |
| PAT-S (FT) | 0.641 | 0.586 | 0.626 | 0.674 | 0.679 | 285 K |
| PAT-S (LP) | 0.643 | 0.598 | 0.617 | 0.676 | 0.679 | 285 K |
| Conv LSTM (Smooth) | 0.655 | 0.583 | 0.639 | 0.700 | 0.698 | 1.75 M |
| PAT Conv-S (FT) | 0.656 | 0.536 | 0.711 | 0.692 | 0.684 | 285 K |
| PAT-L (LP) | 0.662 | 0.495 | 0.721 | 0.713 | 0.720 | 1.99 M |
| PAT Conv-S (LP) | 0.666 | 0.571 | 0.714 | 0.693 | 0.687 | 285 K |
| PAT Conv-M (FT) | 0.668 | 0.552 | 0.705 | 0.705 | 0.712 | 1.00 M |
| CNN-3D | 0.677 | 0.668 | 0.678 | 0.668 | 0.695 | 790 K |
| CNN-3D (Smooth) | 0.680 | 0.671 | 0.675 | 0.682 | 0.692 | 790 K |
| **PAT Conv-M (LP)** | **0.680** | 0.597 | 0.707 | 0.703 | 0.714 | 1.00 M |
| **PAT-M (FT)** | **0.690** | 0.661 | 0.704 | 0.684 | 0.710 | 1.00 M |
| **PAT Conv-L (LP)** | **0.694** | 0.674 | 0.718 | 0.709 | 0.674 | 1.99 M |
| **PAT Conv-L (FT)** | **0.695** | 0.677 | 0.717 | 0.710 | 0.675 | 1.99 M |
| **PAT-L (FT)** | **0.700** | 0.651 | 0.720 | 0.710 | 0.721 | 1.99 M |
| **PAT-M (LP)** | **0.702** | 0.698 | 0.702 | 0.699 | 0.710 | 1.00 M |

***Supplemental Table 2 Evaluating models predicting SSRI usage from actigraphy.*** *In this dataset, the input is actigraphy, and the label indicates whether that participant is taking SSRIs. Each model is trained on dataset sizes "500", "1,000", "2,500", and "5,769", (seen in the columns) and evaluated using AUC on a held-out test set of 2,000 participants. The "Avg AUC" represents the averaged AUC scores across each training dataset size. If the model name has "smoothing" after it, it denotes that it was trained on smoothed data. LP stands for linear probing, and FT stands for end-to-end finetuning. An underline indicates the best baseline model. A bolded PAT model suggests that it performed better than the best baseline, and a bolded and underlined PAT indicates the model with the best performance. PATs outperform the baseline models in every dataset size in this task.*

**Supplemental Table 3. Evaluating models predicting if a participant has or once had a sleep disorder from actigraphy.**

| MODEL | Avg Score* | n=500 | n=1000 | n=2500 | n=3429 | Params |
|---|---|---|---|---|---|---|
| LSTM | 0.494 | 0.480 | 0.490 | 0.509 | 0.499 | 15 K |
| LSTM (Smooth) | 0.506 | 0.486 | 0.511 | 0.489 | 0.540 | 15 K |
| Wavelet Transform | 0.529 | 0.525 | 0.510 | 0.544 | 0.539 | 10 K |
| CNN-1D (Smooth) | 0.558 | 0.556 | 0.540 | 0.570 | 0.566 | 10 K |
| CNN-1D | 0.563 | 0.571 | 0.545 | 0.568 | 0.569 | 10 K |
| PAT-S (FT) | 0.587 | 0.584 | 0.546 | 0.605 | 0.612 | 285 K |
| PAT-S (LP) | 0.596 | 0.586 | 0.579 | 0.607 | 0.613 | 285 K |
| CNN-3D (Smooth) | 0.605 | 0.600 | 0.597 | 0.601 | 0.621 | 790 K |
| Conv LSTM | 0.606 | 0.591 | 0.608 | 0.602 | 0.623 | 1.75 M |
| CNN-3D | 0.608 | 0.611 | 0.612 | 0.587 | 0.624 | 790 K |
| Conv LSTM (Smooth) | 0.609 | 0.591 | 0.604 | 0.609 | 0.633 | 1.75 M |
| **PAT Conv-S (LP)** | **0.613** | 0.600 | 0.619 | 0.615 | 0.620 | 285 K |
| **PAT Conv-M (FT)** | **0.616** | 0.588 | 0.622 | 0.617 | 0.637 | 1.00 M |
| **PAT Conv-S (FT)** | **0.616** | 0.600 | 0.624 | 0.617 | 0.622 | 285 K |
| **PAT Conv-M (LP)** | **0.616** | 0.587 | 0.621 | 0.618 | 0.637 | 1.00 M |
| **PAT Conv-L (LP)** | **0.627** | 0.614 | 0.632 | 0.626 | 0.636 | 1.99 M |
| **PAT Conv-L (FT)** | **0.631** | 0.624 | 0.633 | 0.630 | 0.637 | 1.99 M |
| **PAT-L (FT)** | **0.632** | 0.633 | 0.644 | 0.613 | 0.638 | 1.00 M |
| **PAT-L (LP)** | **0.634** | 0.631 | 0.644 | 0.621 | 0.639 | 1.00 M |
| **PAT-M (FT)** | **0.641** | 0.625 | 0.647 | 0.639 | 0.652 | 1.99 M |
| **PAT-M (LP)** | **0.641** | 0.624 | 0.647 | 0.640 | 0.652 | 1.99 M |

***Supplemental Table 3 Evaluating models predicting if participant has or once had a sleep disorder from actigraphy.*** *In this dataset, the input is actigraphy, and the label indicates if a participant has or once had a sleep disorder. Each model is trained on dataset sizes "500", "1,000", "2,500", and "3,429", (seen in the columns) and evaluated using AUC on a held-out test set of 2,000 participants. The "Avg AUC" represents the averaged AUC scores across each training dataset size. If the model name has "smoothing" after it, it denotes that it was trained on smoothed data. LP stands for linear probing, and FT stands for end-to-end finetuning. An underline indicates the best baseline model. A bolded PAT model suggests that it performed better than the best baseline, and a bolded and underlined PAT indicates the model with the best performance. PATs outperform the baseline models in every dataset size in this task.*

**Supplemental Table 4. Evaluating models in predicting sleep abnormality from actigraphy.**

| MODEL | Avg Score* | n=500 | n=1000 | n=2500 | n=3429 | Params |
|---|---|---|---|---|---|---|
| LSTM | 0.513 | 0.500 | 0.498 | 0.532 | 0.524 | 15 K |
| LSTM (Smooth) | 0.515 | 0.522 | 0.524 | 0.493 | 0.522 | 15 K |
| CNN-1D (Smooth) | 0.519 | 0.478 | 0.513 | 0.534 | 0.550 | 10 K |
| Wavelet Transform | 0.525 | 0.503 | 0.525 | 0.547 | 0.524 | 10 K |
| CNN-1D | 0.534 | 0.501 | 0.548 | 0.549 | 0.538 | 10 K |
| PAT-S (FT) | 0.555 | 0.527 | 0.516 | 0.610 | 0.568 | 285 K |
| PAT-S (LP) | 0.565 | 0.531 | 0.558 | 0.607 | 0.565 | 285 K |
| PAT Conv-S (LP) | 0.571 | 0.512 | 0.596 | 0.610 | 0.564 | 285 K |
| PAT Conv-S (FT) | 0.573 | 0.506 | 0.604 | 0.615 | 0.568 | 285 K |
| Conv LSTM (Smooth) | 0.579 | 0.518 | 0.609 | 0.592 | 0.598 | 1.75 M |
| Conv LSTM | 0.585 | 0.558 | 0.607 | 0.586 | 0.589 | 1.75 M |
| CNN-3D | 0.606 | 0.596 | 0.632 | 0.547 | 0.650 | 790 K |
| <u>CNN-3D (Smooth)</u> | <u>0.615</u> | 0.588 | 0.618 | 0.628 | 0.625 | 790 K |
| **PAT Conv-M (FT)** | **0.627** | 0.591 | 0.624 | 0.649 | 0.644 | 1.00 M |
| **PAT Conv-M (LP)** | **0.632** | 0.599 | 0.632 | 0.649 | 0.647 | 1.00 M |
| **PAT-M (LP)** | **0.641** | 0.583 | 0.653 | 0.661 | 0.665 | 1.00 M |
| **PAT-M (FT)** | **0.641** | 0.585 | 0.653 | 0.661 | 0.666 | 1.00 M |
| **PAT Conv-L (LP)** | **0.659** | 0.614 | 0.661 | 0.676 | 0.685 | 1.99 M |
| **PAT Conv-L (FT)** | **0.659** | 0.616 | 0.661 | 0.675 | 0.685 | 1.99 M |
| **PAT-L (LP)** | **0.665** | 0.627 | 0.667 | 0.678 | 0.686 | 1.99 M |
| **<u>PAT-L (FT)</u>** | **<u>0.665</u>** | 0.626 | 0.667 | 0.679 | 0.686 | 1.99 M |

***Supplemental Table 4 Evaluating models in predicting sleep abnormality from actigraphy.*** *In this dataset, the input is actigraphy, and the label indicates whether that participant has sleep abnormalities. Each model is trained on dataset sizes "500", "1,000", "2,500", and "3,429", (seen in the columns) and evaluated using AUC on a held-out test set of 2,000 participants. The "Avg AUC" represents the averaged AUC scores across each training dataset size. If the model name has "smoothing" after it, it denotes that it was trained on smoothed data. LP stands for linear probing, and FT stands for end-to-end finetuning. An underline indicates the best baseline model. A bolded PAT model suggests that it performed better than the best baseline, and a bolded and underlined PAT indicates the model with the best performance. PATs outperform the baseline models in every dataset size in this task.*

## Supplemental Table 5. Evaluating models predicting depression from actigraphy.

| MODEL | Avg Score* | n=500 | n=1000 | n=2500 | n=2800 | Params |
|---|---|---|---|---|---|---|
| LSTM | 0.489 | 0.472 | 0.489 | 0.497 | 0.497 | 15 K |
| LSTM (Smooth) | 0.506 | 0.496 | 0.494 | 0.519 | 0.515 | 15 K |
| CNN-1D (Smooth) | 0.517 | 0.461 | 0.540 | 0.537 | 0.528 | 10 K |
| CNN-1D | 0.522 | 0.500 | 0.533 | 0.530 | 0.525 | 10 K |
| Wavelet Transform | 0.523 | 0.550 | 0.531 | 0.512 | 0.500 | 10 K |
| Conv LSTM (Smooth) | 0.547 | 0.476 | 0.561 | 0.573 | 0.580 | 1.75 M |
| Conv LSTM | 0.550 | 0.507 | 0.534 | 0.579 | 0.581 | 1.75 M |
| PAT-M (LP) | 0.557 | 0.488 | 0.597 | 0.564 | 0.577 | 1.00 M |
| PAT-M (FT) | 0.559 | 0.489 | 0.591 | 0.566 | 0.589 | 1.00 M |
| PAT-S (LP) | 0.560 | 0.547 | 0.552 | 0.565 | 0.575 | 285 K |
| PAT-S (FT) | 0.560 | 0.550 | 0.556 | 0.560 | 0.574 | 285 K |
| PAT-L (LP) | 0.582 | 0.495 | 0.595 | 0.618 | 0.620 | 1.99 M |
| CNN-3D (Smooth) | 0.583 | 0.576 | 0.576 | 0.593 | 0.589 | 790 K |
| CNN-3D | 0.586 | 0.587 | 0.580 | 0.598 | 0.580 | 790 K |
| **PAT Conv-S (FT)** | **0.587** | **0.568** | **0.576** | **0.603** | **0.600** | **285 K** |
| **PAT Conv-S (LP)** | **0.587** | **0.567** | **0.575** | **0.604** | **0.603** | **285 K** |
| **PAT-L (FT)** | **0.589** | **0.541** | **0.577** | **0.618** | **0.620** | **1.99 M** |
| **PAT Conv-M (LP)** | **0.589** | **0.556** | **0.584** | **0.611** | **0.605** | **1.00 M** |
| **PAT Conv-M (FT)** | **0.594** | **0.576** | **0.585** | **0.609** | **0.606** | **1.00 M** |
| **PAT Conv-L (FT)** | **0.610** | **0.594** | **0.606** | **0.617** | **0.624** | **1.99 M** |
| **PAT Conv-L (LP)** | **0.611** | **0.594** | **0.606** | **0.618** | **0.625** | **1.99 M** |

***Supplemental Table 5 Evaluating models predicting depression from actigraphy.*** *In this dataset, the input is actigraphy, and the label indicates whether that participant has depression (PHQ-9 scores > 9). Each model is trained on dataset sizes "500", "1,000", "2,500", and "2,800", (seen in the columns) and evaluated using AUC on a held-out test set of 2,000 participants. The "Avg AUC" represents the averaged AUC scores across each training dataset size. If the model name has "smoothing" after it, it denotes that it was trained on smoothed data. LP stands for linear probing, and FT stands for end-to-end finetuning. An underline indicates the best baseline model. A bolded PAT model suggests that it performed better than the best baseline, and a bolded and underlined PAT indicates the model with the best performance. PATs outperform the baseline models in every dataset size in this task.*

**Supplemental Table 6. Finding optimizing mask ratio, data preprocessing, and loss function.**

| (a) MODEL (Size, Masking Ratio) | Avg Score* | n=500 | n=1000 | n=2500 | n=4769 |
|---|---|---|---|---|---|
| Medium 0.50 (LP) | 0.703 | 0.661 | 0.672 | 0.693 | 0.785 |
| Medium 0.50 (FT) | 0.707 | 0.727 | 0.672 | 0.642 | 0.788 |
| Medium 0.25 (LP) | 0.724 | 0.689 | 0.698 | 0.765 | 0.744 |
| Medium 0.25 (FT) | 0.737 | 0.711 | 0.734 | 0.745 | 0.759 |
| Medium 0.75 (FT) | 0.743 | 0.662 | 0.742 | 0.798 | 0.772 |
| Medium 0. 75 (LP) | 0.747 | 0.686 | 0.753 | 0.781 | 0.767 |
| Medium 0.90 (LP) | 0.768 | 0.720 | 0.766 | 0.794 | 0.792 |
| **Medium 0.90 (FT)** | **0.773** | 0.753 | 0.764 | 0.786 | 0.788 |

| (b) MODEL (Size, Smoothing) | Avg Score* | n=500 | n=1000 | n=2500 | n=4769 |
|---|---|---|---|---|---|
| Medium Smooth (LP) | 0.734 | 0.686 | 0.686 | 0.767 | 0.797 |
| Medium Smooth (FT) | 0.741 | 0.732 | 0.682 | 0.756 | 0.792 |
| Medium Raw (LP) | 0.768 | 0.720 | 0.766 | 0.794 | 0.792 |
| **Medium Raw (FT)** | **0.773** | 0.753 | 0.764 | 0.786 | 0.788 |

| (c) MODEL (Size, Loss) | Avg Score* | n=500 | n=1000 | n=2500 | n=4769 |
|---|---|---|---|---|---|
| Medium, MSE MASK (LP) | 0.534 | 0.461 | 0.430 | 0.542 | 0.704 |
| Medium, MSE MASK (FT) | 0.541 | 0.437 | 0.515 | 0.560 | 0.652 |
| Medium, MSE ALL (LP) | 0.768 | 0.720 | 0.766 | 0.794 | 0.792 |
| **Medium, MSE ALL (FT)** | **0.773** | 0.753 | 0.764 | 0.786 | 0.788 |

***Supplemental Table 6 Finding optimizing mask ratio, data preprocessing, and loss function.*** *All models are pretrained and end-to-end fine-tuned (FT) on the benzodiazepine training data. The difference between this table and Table 7 in the manuscript is that we also show linear probing (LP) results. For the experiments, 1,000 participants were removed from the training data to create an evaluation set, and these 1,000 participants are separate from the held-out test set seen in Table 1. The "Avg Score" metric is the average AUC score on the evaluation set after the medium model was trained on dataset sizes "500", "1,000", "2,500", and "4,769". (a) We test a PAT-M pretrained using MSE loss on every data point. We find that a higher mask ratio during pretraining leads to better results. (b) We test PAT-M pretrained on 90% masking and MSE loss on all data and find that smoothing does not improve performance (c) We test a PAT-M with 90% masking and find that MSE on only the masked patches decreases performance.*

## Supplemental Figure 1: Attention Weight Patterns and Daily Activity Trends for a Non-Benzodiazepine Participant

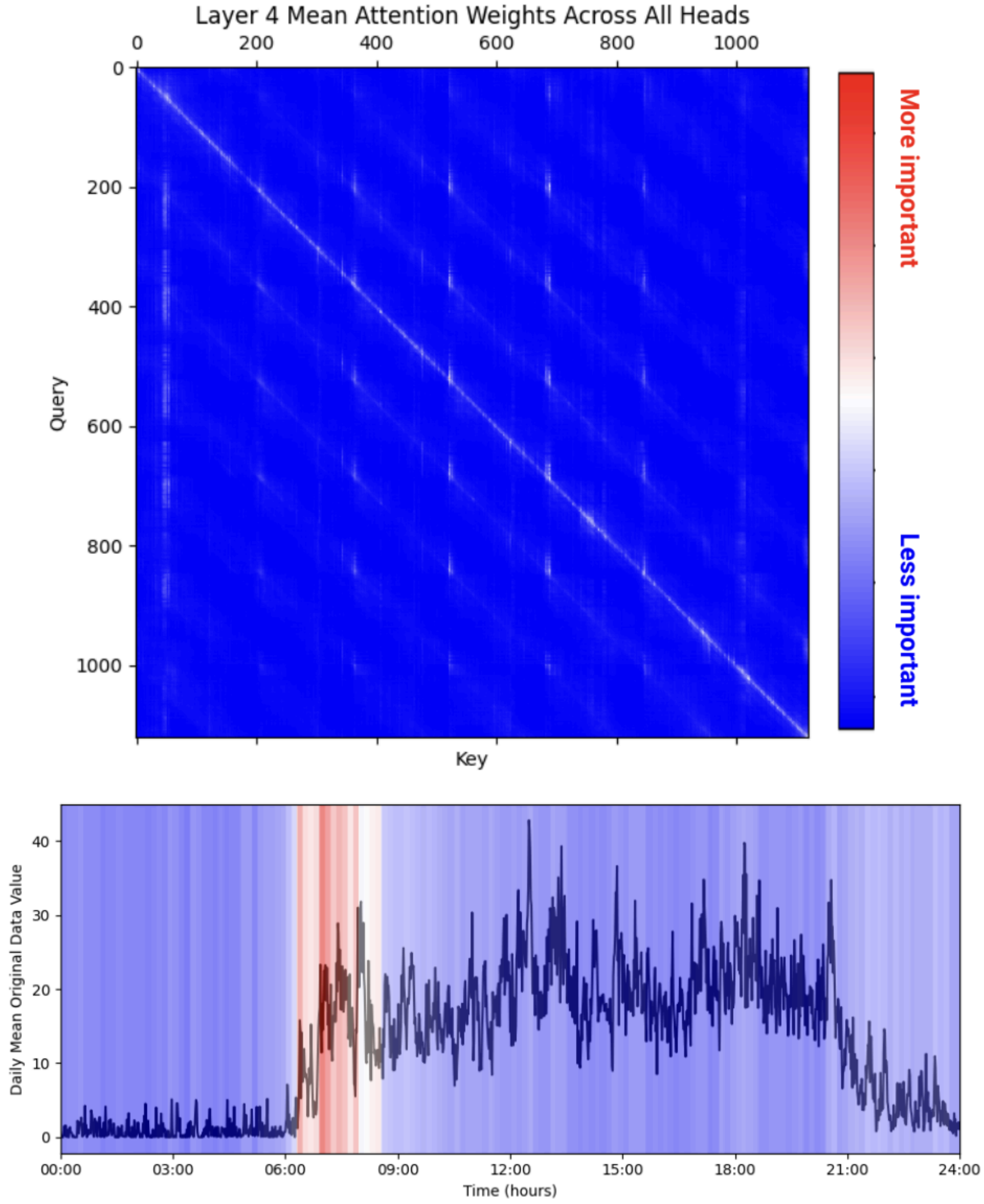


***Supplemental Figure 1. Attention Weight Patterns and Daily Actigraphy Trends for a Non-Benzodiazepine Participant.*** *This figure presents attention weight patterns and daily actigraphy trends for a participant who is not taking benzodiazepines. (Top Panel) The attention weight matrix from Layer 4 of the PAT model across all heads. Bright white dots and the diagonals between them correspond to naturally captured relationships such as consecutive days and hours. (Bottom Panel) Daily mean actigraphy values, with attention weights mapped to activity levels. The model highlights early and sharp wake-up times as important features deciding whether or not a participant takes benzodiazepines.*
